# Comparison of several blood lipid-related indexes in the screening of non-alcoholic fatty liver disease in women: a cross-sectional study in the Pearl River Delta region of southern China

**DOI:** 10.1186/s12876-021-02072-1

**Published:** 2021-12-19

**Authors:** Jingrui Wang, Zhenzhen Su, Yijin Feng, Ruihan Xi, Jiamin Liu, Peixi Wang

**Affiliations:** 1 Institute of Chronic Disease Risks Assessment, School of Nursing and Health, Henan University, Kaifeng, 475004 China; 2 General Practice Center, The Seventh Affiliated Hospital of Southern Medical University, Foshan, 528244 China

**Keywords:** Blood lipid-related indexes, Screening ability, Non-alcoholic fatty liver disease, Women, Cross-sectional study, Southern China

## Abstract

**Background:**

Lipid metabolism disorders play a critical role in the progression of non-alcoholic fatty liver disease (NAFLD). However, the number of studies on the relationships among blood lipid-related indexes and NAFLD is limited, and few studies have emphasized the comparison of blood lipid-related indexes in the same population to identify the optimal index for NAFLD screening. This study aimed to investigate the relationships among several blood lipid-related indexes and NAFLD, and to find the index with the best screening value for NAFLD.

**Methods:**

Based on a general health examination at community health service agencies in the Pearl River Delta region of China in 2015, 3239 women were recruited in this cross-sectional study. The relationships among blood lipid-related indexes and NAFLD were assessed separately by constructing multivariate logistic regression models. Receiver operating characteristic analysis was used to evaluate and compare the screening abilities of the indexes for NAFLD. All data analyses were conducted in SPSS and MedCalc software.

**Results:**

Whether in the crude model or each model adjusted for possible confounding factors, the risk of NAFLD significantly rose with increasing cardiometabolic index (CMI), triglyceride glucose index (TyG), triglycerides (TG) to high-density lipoprotein cholesterol (HDL-C) ratio (TG/HDL-C), total cholesterol (TC) to HDL-C ratio (TC/HDL-C) and low-density lipoprotein (LDL-C) to HDL-C ratio (LDL-C/HDL-C). Moreover, the area under the curve (AUC) of CMI was 0.744, which was better than that of TyG (0.725), TG/HDL-C (0.715), TC/HDL-C (0.650), and LDL-C/HDL-C (0.644) (*P* < 0.001). In addition, the optimal cut-off points were 0.62 for CMI, 8.55 for TyG, 1.15 for TG/HDL-C, 4.17 for TC/HDL-C, and 2.22 for LDL-C/HDL-C.

**Conclusions:**

CMI is easy to obtain, is a recommended index in the screening of NAFLD in women and may be useful for detecting populations that are at high risk of NAFLD.

## Background

Non-alcoholic fatty liver disease (NAFLD) is considered to be the most common liver disease and is the main cause of chronic liver disease, affecting approximately one-quarter of the general population worldwide [1, 2]. There are regional variations in its prevalence. For example, the prevalence of NAFLD is 27.37% in Asia, 24.13% in North America, the highest prevalence is 31.79% in the Middle East and the lowest prevalence is 13.48% in Africa [3]. Estimates suggest that the prevalence of NAFLD continues to be on the rise globally [4, 5]. The spectrum of NAFLD includes simple steatosis, non-alcoholic steatohepatitis (NASH), hepatic fibrosis, and cirrhosis. Among them, NASH has the potential to progress to cirrhosis and hepatocellular carcinoma (HCC), leading to liver transplantation or death among some patients [6]. In addition to the serious consequences that NAFLD may progress, NAFLD can increase the clinical and economic burden on patients worldwide. It has been reported that NAFLD-related HCC is the most rapidly growing cause of liver transplantation in the USA, and in 2014, the total estimated national hospitalization costs to patients with NAFLD reached $19.9 billion [7, 8]. Additionally, the estimated total charges associated with NASH in 2018 were between £5.6 and £10.5 billion in the UK [9]. In China, NAFLD has become an important public health problem with the rapid increase in the number of patients with NAFLD [4]. Moreover, patients with NAFLD have a high prevalence of various comorbidities (e.g., metabolic syndrome [MS], cardiovascular disease and chronic kidney disease, et al.), which lead to increasing hospitalization charges and decreasing quality of life [5, 10–12]. Thus, more attention should be given to NAFLD based on the above characteristics.

The gold standard for the diagnosis of NAFLD is liver biopsy, but it is obviously an expensive and invasive procedure with risks of mortality, bleeding, infection and pain [13]. These issues limit the use of liver biopsy in the screening for NAFLD in the population. As an alternative tool of liver biopsy, liver ultrasonography is widely used for the diagnosis of NAFLD in clinical practice [14, 15]. However, when ultrasound imaging cannot be obtained in some large-scale screening programmes, the use of biomarker panels based on blood samples to identify NAFLD is a good method [16, 17]. Therefore, it is necessary to explore simpler and more effective monitoring indicators as the supplementary tools for ultrasonography, for the risk assessment and early screening of NAFLD.

The inner mechanisms underlying NAFLD are not fully understood [18]. However, animal model tests have shown that dyslipidemia can lead to the occurrence and development of hepatic steatosis [19, 20]. Moreover, alterations in plasma lipoproteins are very common in patients with NAFLD and are closely related to the main complication of NAFLD, cardiovascular disease [21, 22]. Paying attention to the relationship between the lipid profile and NAFLD plays an important role in the overall management of patients with NAFLD. Furthermore, lipid levels in blood samples are easy to measure during routine physical examinations. Thus, the lipid profile may be potential indicators of identifying NAFLD. In recent years, studies have found that several indexes related to blood lipids, such as the triglyceride glucose index (TyG) [23, 24], triglycerides (TG) to high-density lipoprotein cholesterol (HDL-C) ratio (TG/HDL-C) [25, 26], total cholesterol (TC) to HDL-C ratio (TC/HDL-C) [27] and low-density lipoprotein cholesterol (LDL-C) to HDL-C ratio (LDL-C/HDL-C) [28], have significant predictive value for NAFLD, and these combined indexes of blood lipids are better than an isolated lipoprotein in predicting the risk of NAFLD. Additionally, as a new predictor of obesity and blood lipids, the cardiometabolic index (CMI) has been shown to be associated with diabetes, cardiovascular disease and MS [29–32]. Both CMI and NAFLD have relationships with these diseases, but there is limited available evidence suggesting an association of CMI with NAFLD. To our knowledge, only one Chinese study has reported that CMI has good predictive value for NAFLD in patients with type 2 diabetes [33].

Here, we performed a cross-sectional study to explore the relationships among the above blood lipid-related indexes (especially CMI) and NAFLD, and to find the index with the best screening value for NAFLD in women based on health examination data of residents in southern China. Studies have typically used sex as an adjustment variable [27, 34, 35], but we were told that the prevalence of obesity among women (5.9%) was higher than that among men (4.8%) in China [36] and that the prevalence of MS among women (36.8%; 35.6%) was higher than that among men (31.0%; 30.3%) [37, 38]. It is known that the incidence and development of NAFLD are closely related to obesity, and NAFLD is considered to be the hepatic manifestation of MS [39, 40]. Thus, based on the contributions of women to the prevalence of obesity and MS, our intent was to focus on women with NAFLD. Our study can provide a simple and reliable index for the early identification of women with NAFLD.

## Methods

### Participants

In total, 3239 subjects who underwent a general health examination at community health service centers in the Pearl River Delta region of China in 2015 were recruited in this cross-sectional study. The health examination included self-reported medical history, physical examination, instrument-based examination (abdominal ultrasonography, electrocardiogram and chest X-ray) and laboratory examination. Participants eligible for this study were women who were diagnosed with fatty liver by liver ultrasonography. Participants with a history of excessive alcohol consumption (alcohol intake ≥ 20 g/day) or other possible causes of hepatic steatosis (viral or autoimmune) were excluded. In addition, subjects without complete health examination data in Table 1 were also excluded. To protect the confidentiality of personal information of participants, we hid their names and ID card numbers. To protect and promote the health of research subjects, all procedures were performed in accordance with the Declaration of Helsinki, and ethics approval was obtained from the ethics committee of Guangdong Sociological Society. Moreover, informed consent was obtained from all participants.

**Table 1:** Characteristics of the participants with and without NAFLD (N = 3239)

Variables	Total	Non-NAFLD (n = 2527)	NAFLD* (n = 712)	*P*
Age (years)	57.65 ± 12.53	57.23 ± 12.90	59.11 ± 11.04	< 0.001
*Medical history*				
Hypertension, n (%)				0.138
Yes	1640 (50.6%)	1262 (77.0%)	378 (23.0%)	
No	1599 (49.4%)	1265 (79.1%)	334 (20.9%)	
Diabetes, n (%)				0.012
Yes	394 (12.2%)	288 (73.1%)	106 (26.9%)	
No	2845 (87.8%)	2239 (78.7%)	606 (21.3%)	
*Anthropometric indexes*				
SBP (mmHg)	130.08 ± 18.58	128.93 ± 18.56	134.16 ± 18.07	< 0.001
DBP (mmHg)	81.15 ± 10.89	80.44 ± 10.85	83.66 ± 10.69	< 0.001
BMI (kg/m^2^)	24.40 ± 3.64	23.61 ± 3.29	27.20 ± 3.42	< 0.001
*Routine blood test*				
RBC (× 10^12^/L)	4.15 ± 1.06	4.13 ± 1.18	4.24 ± 0.42	0.020
WBC (× 10^9^/L)	6.29 ± 1.58	6.12 ± 1.52	6.89 ± 1.67	< 0.001
Hb (g/L)	122.73 ± 11.09	121.73 ± 11.01	126.26 ± 10.68	< 0.001
PLT (× 10^9^/L)	222.53 ± 55.20	219.71 ± 53.77	232.53 ± 58.99	< 0.001
*Glucose level*				
FPG (mmol/L)	4.73 (4.30–5.31)	4.68 (4.27–5.19)	4.97 (4.49–5.87)	< 0.001
HbAlc (%)	5.30 (5.00–5.60)	5.30 (5.00–5.60)	5.40 (5.10–5.90)	< 0.001
*Liver function test*				
ALT (U/L)	21.00 (16.00–27.00)	19.00 (16.00–24.00)	27.00 (21.00–36.00)	< 0.001
AST (U/L)	22.00 (19.00–26.00)	22.00 (19.00–25.00)	24.00 (21.00–29.75)	< 0.001
ALP (U/L)	80.88 ± 23.45	79.79 ± 24.01	84.75 ± 20.91	< 0.001
GGT (U/L)	24.00 (19.00–33.00)	23.00 (19.00–30.00)	33.00 (25.00–45.00)	< 0.001
TBIL (μmol/L)	12.17 ± 5.22	12.32 ± 5.53	11.65 ± 3.89	0.003
*Renal function test*				
Scr (μmol/L)	78.40 (74.90–80.00)	78.20 (74.70–80.00)	78.90 (75.50–80.98)	< 0.001
BUN (mmol/L)	4.90 (4.00–5.90)	4.90 (4.00–5.90)	5.00 (4.10–6.00)	0.047
UA (μmol/L)	328.79 ± 98.16	317.88 ± 97.42	367.54 ± 90.74	< 0.001
*Blood lipid test*				
TC (mmol/L)	5.20 ± 1.42	5.14 ± 1.05	5.41 ± 2.28	0.002
TG (mmol/L)	1.34 (0.96–1.92)	1.22 (0.90–1.71)	1.84 (1.35–2.59)	< 0.001
HDL-C (mmol/L)	1.35 ± 0.33	1.38 ± 0.34	1.24 ± 0.28	< 0.001
LDL-C (mmol/L)	2.85 ± 1.04	2.81 ± 0.95	3.01 ± 1.30	< 0.001
*Blood lipid-related indexes*				
CMI	0.78 ± 0.91	0.67 ± 0.82	1.17 ± 1.08	< 0.001
TyG	8.61 ± 0.63	8.50 ± 0.58	8.99 ± 0.65	< 0.001
TG/HDL-C	1.43 ± 1.71	1.26 ± 1.61	2.02 ± 1.92	< 0.001
TC/HDL-C	4.05 ± 1.44	3.90 ± 1.22	4.55 ± 1.97	< 0.001
LDL-C/HDL-C	2.23 ± 0.97	2.14 ± 0.85	2.54 ± 1.28	< 0.001

Data are presented as the n (%), mean ± standard deviation (SD) or median (interquartile range)

*NAFLD* non-alcoholic fatty liver disease, *SBP* systolic blood pressure, *DBP* diastolic blood pressure, *BMI* body mass index, *RBC* red blood cell, *WBC* white blood cell, *Hb* hemoglobin, *PLT* platelet, *FPG* fasting plasma glucose, *HbAlc* glycated hemoglobin, *ALT* alanine aminotransferase, *AST* aspartate aminotransferase, *ALP* alkaline phosphatase, *GGT* γ-glutamyl transpeptidase, *TBIL* total bilirubin, *Scr* serum creatinine, *BUN* blood urea nitrogen, *UA* uric acid, *TC* total cholesterol, *TG* triglyceride, *HDL-C* high-density lipoprotein cholesterol, *LDL-C* low-density lipoprotein cholesterol, *CMI* cardiometabolic index, *TyG* triglyceride glucose

^*^Mild NAFLD: 316 (44.4%); moderate NAFLD: 110 (15.4%); severe NAFLD: 15 (2.1%); unreported severity: 271 (38.1%)

### Data collection, measurements and quality control

Demographic data (sex, age) and information on health-related behaviours (smoking, drinking), history of medical conditions diagnosed by doctors (e.g., hypertension, diabetes, hepatitis), and family history were obtained from self-reported questionnaires completed under the guidance of trained investigators.

The physical examination included measurements of weight, height, waist circumference (WC) and blood pressure. Weight and height were measured by weight and height measuring instruments when participants were dressed in light clothes without shoes. WC was measured at the midpoint between the lowest rib and the iliac crest in the standing position with an inelastic soft ruler as the participant remained relaxed. The subject assumed the sitting position, and the right arm was uncovered or placed in light clothes on the table so that the elbow and heart were at the same level. Then, the examiner used an electronic sphygmomanometer produced by Omron company to measure systolic blood pressure (SBP) and diastolic blood pressure (DBP).

After an overnight fast, venous blood samples were collected by trained medical staff and analyzed with a blood analyzer (Mindray BC-2900, Shenzhen, China) and an automatic biochemical analyzer (Mindray BS-420, Shenzhen, China). The laboratory parameters included in this study were red blood cell (RBC), white blood cell (WBC), hemoglobin (Hb), platelet (PLT), fasting plasma glucose (FPG), glycated hemoglobin (HbAlc), alanine aminotransferase (ALT), aspartate aminotransferase (AST), alkaline phosphatase (ALP), γ-glutamyl transpeptidase (GGT), total bilirubin (TBIL), serum creatinine (Scr), blood urea nitrogen (BUN), uric acid (UA), total cholesterol (TC), triglyceride (TG), high-density lipoprotein cholesterol (HDL-C) and low-density lipoprotein cholesterol (LDL-C).

A reasonable number of medical examiners was arranged, and they were trained in and carried out the standard operating procedure. In addition, there were full-time staff to supervise and verify the authenticity of the data.

### Diagnosis of NAFLD

All participants in this study received abdominal liver ultrasonography (Mindray DC-6, Shenzhen, China) performed by professional ultrasound physicians according to the characteristics of the liver anterior echo enhancement (“bright liver”), far-field echo attenuation and unclear intrahepatic duct structure. Participants who consumed more than 20 g/day of alcohol or were diagnosed with viral or autoimmune hepatitis were excluded [41]. The ultrasonic diagnosis criteria were in accordance with guidelines for the diagnosis and treatment of NAFLD as follows [42]: (1) liver anterior echo enhancement (stronger than that in the kidney and spleen) and far-field echo attenuation; (2) unclear intrahepatic duct structure; (3) mild to moderate hepatomegaly with blunted borders; (4) color Doppler flow imaging showing reduced intrahepatic blood flow signal but normal distribution of blood flow; and (5) unclear or incomplete echo of the capsule of the right lobe of the liver and diaphragm. The diagnostic criteria of mild fatty liver are item 1 and any one of items 2–4; the diagnostic criteria of moderate fatty liver are item 1 and any two items of items 2–4; The diagnostic criteria of severe fatty liver are items 1 and 5 and any two of items 2–4.

### Derived variables

Body mass index (BMI) was calculated with the following formula: BMI = weight (kg)/height (m)^2^.

The waist-to-height ratio (WHtR) was calculated with the following formula: WHtR = WC (cm)/height (cm).

CMI was calculated with the following formula [29]: CMI = TG/HDL-C × WHtR.

TyG was calculated with the following formula [23]: TyG = ln (TG [mg/dL] × FPG [mg/dL]/2).

### Statistical analysis

Data analyses were conducted in SPSS version 23.0 (SPSS Inc, Chicago, IL) and MedCalc version 19.4.1 (MedCalc Software, Ostend, Belgium) software. Continuous variables are presented as the mean ± standard deviation (SD) for normal distributions or the median (interquartile range) for non-normal distributions. Categorical variables are expressed as frequencies (percentages). For between-group comparisons, Student’s *t*-test (normal distribution) or the Mann–Whitney U test (non-normal distribution) was used for continuous data, and the chi-square test was used for categorical data. The relationships among blood lipid-related indexes and the risk of NAFLD were assessed respectively by constructing multivariate logistic regression models with indexes as categorical variables (divided into four groups according to quartiles) and continuous variables (standardized using z-score normalization). The following four models were created: the crude model, with no adjustments; model I, with adjustments for age and medical history (hypertension, diabetes); model II, with adjustments for model I plus anthropometric indexes (SBP, DBP, and BMI); and model III, with adjustments for model II plus laboratory parameters (RBC, WBC, Hb, PLT, FPG, HbA1c, ALT, AST, ALP, GGT, TBIL, Scr, BUN and UA). The odds ratios (ORs) and 95% confidence intervals (CIs) were also calculated. The area under the curve (AUC) of the receiver operating characteristic (ROC) analysis was used to evaluate the abilities of blood lipid-related indexes to screen for NAFLD (ideal screening tools should have AUC > 0.7), and the highest Youden’s index (sensitivity + specificity − 1) was used to determine the optimal cut-off point of the index to screen for NAFLD. MedCalc software was used to compare different ROC curves. The statistical significance level was set at α = 0.05 (two-tailed).

## Results

### Characteristics of participants

The age, self-reported medical history, physical examination and laboratory examination data of participants are summarized in Table 1. A total of 3239 women were included, with ages ranging from 19 to 93 years (57.65 ± 12.53 years), and the prevalence of NAFLD was 22.0% in our study. Compared with the non-NAFLD group, the women with NAFLD were older and had higher SBP, DBP, BMI, RBC, WBC, Hb, PLT, FPG, HbAlc, ALT, AST, ALP, GGT, Scr, BUN, UA, TC, TG, LDL-C, CMI, TyG, TG/HDL-C, TC/HDL-C, and LDL-C/HDL-C values (*P* < 0.05). However, the levels of TBIL and HDL-C were higher in the control group (*P* < 0.05). The prevalence of NAFLD was higher in the population with diabetes (*P* < 0.05), but no significant difference was observed in the hypertensive population.

### Association between blood lipid-related indexes and NAFLD

**Fig. 1:**
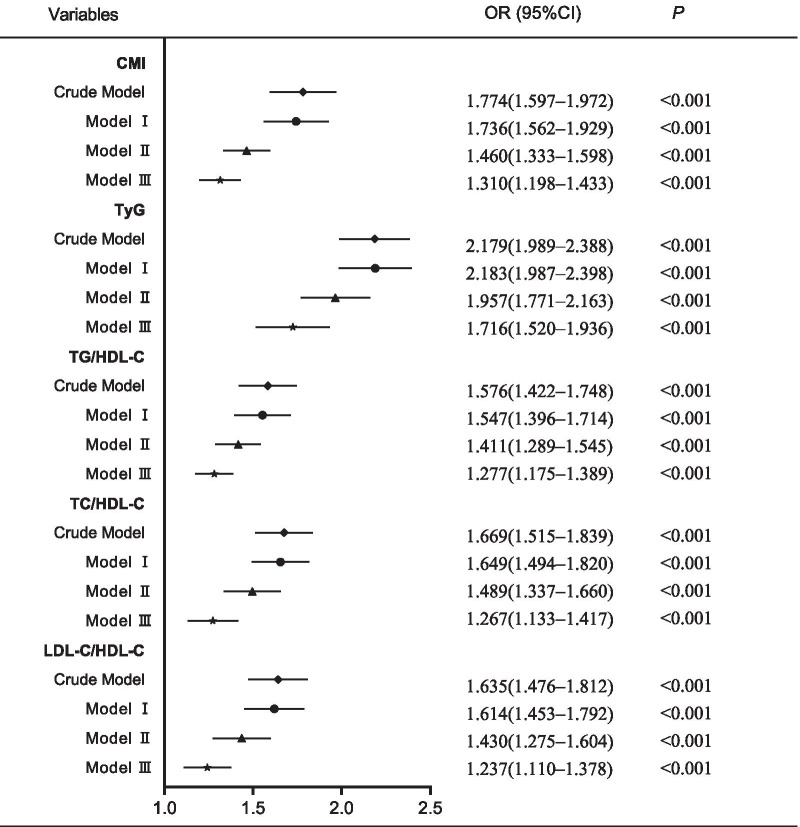
The relationships between blood lipid-related indexes and the risk of NAFLD. These indexes were regarded as continuous variables in the multivariate logistic regression models in this figure. Because the indexes were converted to z scores in the multivariate logistic regression model, OR increase with each 1-unit increase in SD for every index. Crude model: unadjusted; Model I: adjusted for age, hypertension, diabetes; Model II: adjusted for Model I plus SBP, DBP, and BMI; Model III: adjusted for model II plus RBC, WBC, Hb, PLT, FPG, HbA1c, ALT, AST, ALP, GGT, TBIL, Scr, BUN and UA

Table 2 and Fig. 1 present the relationships between NAFLD and blood lipid-related indexes when they were analyzed as categorical variables and continuous variables, respectively. As shown in Table 2, whether in the crude model or any of the models adjusted for possible confounding factors (model I, II or III), the risk of NAFLD significantly rose with increasing CMI quartiles (*P* < 0.001). Additionally, with increasing TyG, TG/HDL-C, TC/HDL-C or LDL-C/HDL-C quartiles, the corresponding OR value also increased gradually. The relationships between the risk of NAFLD and blood lipid-related indexes were also verified in the above model analyses when these indexes were analyzed as continuous variables. Based on z-score standardization, for each 1-unit increase in SD for CMI, TyG, TG/HDL-C, TC/HDL-C or LDL-C/HDL-C, the risk of NAFLD increased by 31.0% (OR: 1.310, 95% CI 1.198–1.433), 71.6% (OR: 1.716, 95% CI 1.520–1.936), 27.7% (OR: 1.277, 95% CI 1.175–1.389), 26.7% (OR: 1.267, 95% CI 1.133–1.417), and 23.7% (OR: 1.237, 95% CI 1.110–1.378), respectively, in model III (all *P* < 0.001). The results indicated that CMI, TyG, TG/HDL-C, TC/HDL-C and LDL-C/HDL-C were related to NAFLD.

**Table 2:** Association of blood lipid-related indexes with the risk of NAFLD in multivariate logistic regression models

Variables	Quartile; OR (95% CI)				
	Q1	Q2	Q3	Q4	*P*
*CMI*					
Crude model	Reference	3.629 (2.475–5.320)	6.978 (4.839–10.063)	16.036 (11.211–22.937)	< 0.001
Model I	Reference	3.574 (2.436–5.244)	6.829 (4.729–9.863)	15.545 (10.844–22.282)	< 0.001
Model II	Reference	2.324 (1.556–3.469)	3.634 (2.471–5.345)	7.519 (5.154–10.969)	< 0.001
Model III	Reference	2.224 (1.464–3.377)	2.902 (1.935–4.353)	5.159 (3.451–7.713)	< 0.001
*TyG*					
Crude model	Reference	2.418 (1.725–3.389)	4.156 (3.015–5.730)	10.883 (7.990–14.823)	< 0.001
Model I	Reference	2.386 (1.701–3.346)	4.052 (2.934–5.595)	10.718 (7.840–14.652)	< 0.001
Model II	Reference	2.163 (1.511–3.095)	2.810 (1.994–3.960)	7.309 (5.245–10.187)	< 0.001
Model III	Reference	1.906 (1.316–2.762)	2.249 (1.571–3.221)	4.780 (3.320–6.880)	< 0.001
*TG/HDL-C*					
Crude model	Reference	2.502 (1.783–3.510)	5.157 (3.749–7.093)	9.301 (6.806–12.710)	< 0.001
Model I	Reference	2.459 (1.752–3.451)	5.002 (3.634–6.886)	8.957 (6.547–12.254)	< 0.001
Model II	Reference	1.932 (1.346–2.771)	3.229 (2.297–4.540)	5.749 (4.119–8.024)	< 0.001
Model III	Reference	1.812 (1.246–2.636)	2.528 (1.766–3.619)	3.886 (2.725–5.542)	< 0.001
*TC/HDL-C*					
Crude model	Reference	1.867 (1.401–2.488)	2.917 (2.217–3.838)	4.299 (3.288–5.620)	< 0.001
Model I	Reference	1.852 (1.388–2.471)	2.874 (2.179–3.790)	4.195 (3.193–5.511)	< 0.001
Model II	Reference	1.394 (1.018–1.907)	1.888 (1.396–2.554)	2.968 (2.208–3.990)	< 0.001
Model III	Reference	1.341 (0.962–1.869)	1.584 (1.150–2.182)	2.119 (1.543–2.908)	< 0.001
*LDL-C/HDL-C*					
Crude model	Reference	1.693 (1.275–2.250)	2.822 (2.156–3.694)	3.852 (2.960–5.015)	< 0.001
Model I	Reference	1.678 (1.262–2.232)	2.792 (2.128–3.663)	3.786 (2.894–4.954)	< 0.001
Model II	Reference	1.323 (0.968–1.809)	1.973 (1.463–2.659)	2.799 (2.086–3.757)	< 0.001
Model III	Reference	1.236 (0.889–1.720)	1.637 (1.194–2.245)	1.960 (1.432–2.681)	< 0.001

Crude model: unadjusted; Model I: adjusted for age, hypertension, diabetes; Model II: adjusted for model I plus SBP, DBP, and BMI; Model III: adjusted for model II plus RBC, WBC, Hb, PLT, FPG, HbA1c, ALT, AST, ALP, GGT, TBIL, Scr, BUN and UA

*OR* odds ratio, *CI* confidence interval

### The ROC analysis of blood lipid-related indexes in the screening of NAFLD

**Fig. 2:**
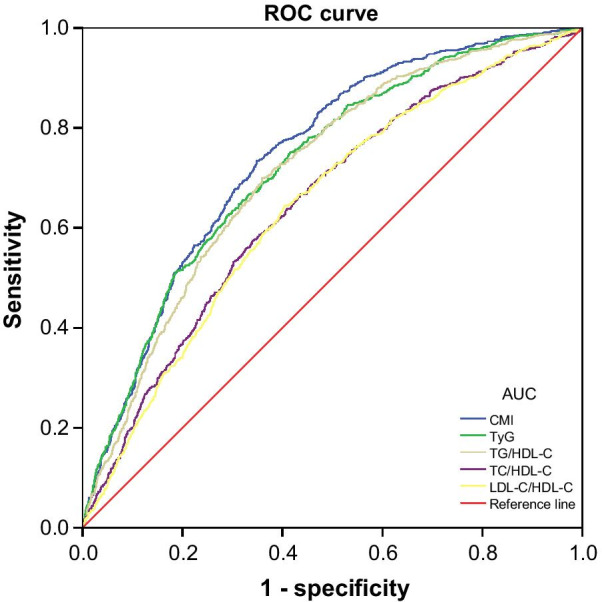
ROC curves of different blood lipid-related indexes in the screening of NAFLD in women

The ROC curves of blood lipid-related indexes were analyzed and compared to explore the screening value and optimal cut-off points for identifying NAFLD and to determine the most notable screening index. ROC curves are presented in Fig. 2. As shown in Table 3, the AUCs of CMI, TyG, TG/HDL-C, TC/HDL-C, and LDL-C/HDL-C were 0.744 (95% CI 0.724–0.763), 0.725 (95% CI 0.705–0.746), 0.715 (95% CI 0.695–0.735), 0.650 (95% CI 0.627–0.672), and 0.644 (95% CI 0.622–0.666) respectively, while the optimal cut-off points were 0.62 (sensitivity = 0.74, specificity = 0.65), 8.55 (sensitivity = 0.75, specificity = 0.58), 1.15 (sensitivity = 0.70, specificity = 0.64), 4.17 (sensitivity = 0.57, specificity = 0.66), and 2.22 (sensitivity = 0.64, specificity = 0.60) respectively for women. The above results suggested that these indexes may be effective in the early screening of NAFLD risk in women, and it is worth noting that the ability of CMI to detect NAFLD was significantly better than that of the other indexes mentioned above (*P* < 0.001) (Table 4).

**Table 3:** ROC curve analysis of the blood lipid-related indexes in the screening of NAFLD

	Cut-off	Sensitivity	Specificity	Youden’s index	AUC (95% CI)
CMI	0.62	0.74	0.65	0.39	0.744 (0.724–0.763)
TyG	8.55	0.75	0.58	0.34	0.725 (0.705–0.746)
TG/HDL-C	1.15	0.70	0.64	0.34	0.715 (0.695–0.735)
TC/HDL-C	4.17	0.57	0.66	0.23	0.650 (0.627–0.672)
LDL-C/HDL-C	2.22	0.64	0.60	0.24	0.644 (0.622–0.666)

*ROC* receiver operating characteristic, *AUC* area under the curve

**Table 4:** Statistical comparison of the effectiveness of different ROC curves

Variables	AUC (95% CI)	*P*				
		CMI	TyG	TG/HDL-C	TC/HDL-C	LDL-C/HDL-C
CMI	0.744 (0.724–0.763)	1	< 0.001	< 0.001	< 0.001	< 0.001
TyG	0.725 (0.705–0.746)		1	0.037	< 0.001	< 0.001
TG/HDL-C	0.715 (0.694–0.735)			1	< 0.001	< 0.001
TC/HDL-C	0.649 (0.627–0.672)				1	0.076
LDL-C/HDL-C	0.644 (0.621–0.666)					1

*ROC* receiver operating characteristic

## Discussion

In recent years, an increasing number of researchers have realized that developing effective, simple and inexpensive tools for identifying NAFLD is a considerably interesting subject [43, 44], but few studies have compared these tools in the same population to identify the best tool for screening of NAFLD. In the current study, several indexes related to blood lipids were analyzed and compared in the screening of NAFLD. We found that CMI, TyG, TG/HDL-C, TC/HDL-C, and LDL-C/HDL-C were risk factors for NAFLD in models with or without adjustments. Additionally, ROC curve analysis indicated that CMI, TyG, and TG/HDL-C could be used to screen for NAFLD effectively (AUC > 0.7), but each of the other two indexes had a poor ability to detect NAFLD (0.5 < AUC < 0.7). Compared with TyG, TG/HDL-C, TC/HDL-C, or LDL-C/HDL-C, CMI was the better index for NAFLD screening in women because it had the larger AUC (*P* < 0.001), which was similar to the results of logistic regression analysis. In the logistic regression models we constructed, CMI had the highest OR values both before and after adjusting for confounding factors when CMI was used as a categorical variable. However, TyG had the highest OR values in the four models when indexes were used as continuous variables.

NAFLD is associated with lipid metabolism disorders [45]. Blood lipid levels can reflect systemic lipid metabolism. Thus, blood lipid-related indexes can be used to screen for NAFLD. The simple and effective lipid profile can be used as "early warning indicators" for the early screening of NAFLD, and people with scores higher than the optimal cut-off point of the indicators (suspected hepatic steatosis disease) can be urged to have a further check-up as soon as possible.

CMI was measured as the product of TG/HDL-C and WHtR. TG/HDL-C is a useful alternative indicator of insulin resistance (IR), and IR plays an important role in the progression of NAFLD [46–49]. IR can promote hepatic lipid deposition by increasing lipolysis of adipocytes and free fatty acids [50]. Regarding TG/HDL-C, our study indicated that it has screening value for NAFLD, and several studies have confirmed the relationship between TG/HDL-C and NAFLD. In a cross-sectional study of 18,061 healthy individuals, researchers have shown that TG/HDL-C may be used as a surrogate for NAFLD [26]. A cohort study of Japanese population revealed that TG/HDL-C could predict the incidence of fatty liver [25]. In addition, studies have shown that obesity, especially abdominal obesity, is associated with NAFLD [39, 51]. WHtR, as an indicator of abdominal obesity, can detect the risk of NAFLD [52, 53]. In previous studies, CMI was associated with a variety of metabolic diseases including obesity and type 2 diabetes, but few studies evaluated the relationship between CMI and the risk of NAFLD [29, 54]. Our study filled this knowledge gap and provided a basis for further research. Our results suggested that the AUC of CMI was 0.744 and that the optimal cut-off point was 0.62. In a study in China [33], the AUC of CMI for screening NAFLD was 0.698, and the optimal cut-off point was 0.694 among women. The small differences may be because the populations were different. They explored the association between CMI and NAFLD in a population of patients with type 2 diabetes, while the subjects in our study were women who underwent health examinations. CMI was better than TG/HDL-C in our study, and another study found that CMI was better than WHtR [33]. CMI takes into account the index of blood lipids and obesity; thus, CMI is reliable and recommended by us as a screening index of NAFLD. It may be beneficial to reduce the CMI of women with NAFLD.

Currently, among many available predictive models for detecting NAFLD, Fatty Liver Index (FLI) is a common and non-invasive score with good accuracy to detect fatty liver, which has been confirmed in many studies [55–57]. The use of FLI can help medical service providers screen NAFLD. However, the calculation method of FLI [55] is more complex than that of CMI (the calculation of FLI requires the use of scientific calculators or computers), and it is not easy for the public to calculate FLI. Particularly, in China’s rural areas with severe health workforce challenges and limited use of appropriate care [58] (more than one third of the Chinese population lived in rural areas as of 2020 [59]), the simplicity of surrogate markers are very important to guide the population to early detection of NAFLD by themselves. Thus, based on these, CMI may be easier to apply than FLI.

In addition to CMI and TG/HDL-C, we found that TyG can also be used as a useful screening index of NAFLD. Similar to our study, a retrospective cohort study involving 46,693 elderly individuals in China reported that a higher TyG index was related to a greater risk of NAFLD [23]. Moreover, the screening power of TC/HDL-C and LDL-C/HDL-C for NAFLD was similar; although they all had certain abilities, they were not good indexes in screening for NAFLD. The AUC of TC/HDL-C was 0.650 in women, which was in accordance with previous studies of the association between TC/HDL-C and NAFLD. Research by Wu KT [60] and his colleague showed that adults with high TC/HDL-C had a higher risk for NAFLD, and Ren et al. [27] found that the AUC of TC/HDL-C was 0.645 for predicting NAFLD in a Jinchang cohort study. However, there were no data on the ability of TC/HDL-C to detect NAFLD in women in Ren’s study. The AUC of LDL-C/HDL-C was 0.644 in our study. In a nonobese population based on a 5-year longitudinal cohort study, Zou et al. [28] found that the predictive value of LDL-C/HDL-C for the risk of new-onset NAFLD was 0.671, which was slightly higher than our AUC, possibly due to different study designs and populations. In short, the discrimination abilities of TC/HDL-C and LDL-C/HDL-C were lower than those of CMI, TyG and TG/HDL-C. This finding may be because the abnormal accumulation of TG in the liver was most closely associated with the risk of NAFLD compared with other blood lipid indexes (TC, HDL-C and LDL-C) [61], and TG was a component of CMI, TyG and TG/HDL-C, but was not included in TC/HDL-C or LDL-C/HDL-C.

The strengths of this study are as follows. First, health examinations were carried out by professional medical staff, which helped to reduce error in the examination data. Second, we used rigorous statistical methods, including the construction of different models, to analyze the relationships between blood lipid-related indexes and NAFLD. However, there are several limitations that should be noted. First, this study was a cross-sectional study, and a prospective cohort study is warranted to examine the cause-effect relationship between blood lipid-related indexes and NAFLD. Second, subjects in the present study were from communities in southern China; thus, further in-depth studies should be performed in different countries and regions before applying our findings to people with NAFLD in other countries and regions. Third, we used abdominal liver ultrasonography rather than liver biopsy to diagnose NAFLD. Finally, besides lipid profile, more potentially significant indexes should be explored in future studies to identify the presence of NAFLD, and we also hope that more potential confounding factors (e.g., physical activity and diet) can be adjusted in future longitudinal follow-up data to further verify the relationship between CMI and NAFLD.

## Conclusions

In conclusion, the abilities of CMI, TyG, TG/HDL-C, TC/HDL-C, and LDL-C/HDL-C for detecting NAFLD are different. In our study, CMI was easy to obtain and is a recommended index in the screening of NAFLD in women. Moreover, the optimal cut-off point of CMI was 0.62 in women. Our findings may provide help towards identifying which populations are at high risk of NAFLD, to help medical workers and individuals prevent NAFLD and intervene in its progression as early as possible.

## Data Availability

The dataset used during the current study is available from the corresponding author on reasonable request.

## Abbreviations

NAFLD: Non-alcoholic fatty liver disease
NASH: Non-alcoholic steatohepatitis
HCC: Hepatocellular carcinoma
TyG: Triglyceride glucose
TG: Triglycerides
HDL-C: High-density lipoprotein cholesterol
TG/HDL-C: Triglycerides to high-density lipoprotein cholesterol ratio
TC: Total cholesterol
TC/HDL-C: Total cholesterol to high-density lipoprotein cholesterol ratio
LDL-C: Low density lipoprotein cholesterol
LDL-C/HDL-C: Low density lipoprotein cholesterol to high-density lipoprotein cholesterol ratio
CMI: Cardiometabolic index
MS: Metabolic syndrome
WC: Waist circumference
SBP: Systolic blood pressure
DBP: Diastolic blood pressure
RBC: Red blood cell
WBC: White blood cell
Hb: Hemoglobin
PLT: Platelet
FPG: Fasting plasma glucose
HbAlc: Glycated hemoglobin
ALT: Alanine aminotransferase
AST: Aspartate aminotransferase
ALP: Alkaline phosphatase
GGT: γ-Glutamyl transpeptidase
TBIL: Total bilirubin
Scr: Serum creatinine
BUN: Blood urea nitrogen
UA: Uric acid
BMI: Body mass index
WHtR: Waist-to-height ratio
SD: Standard deviation
OR: Odds ratio
CI: Confidence interval
AUC: Area under the curve
ROC: Receiver operating characteristic
IR: Insulin resistance
FLI: Fatty liver index
